# Nitric Oxide and a Conditioned Medium Affect the Hematopoietic Development in a Microfluidic Mouse Embryonic Stem Cell/OP9 Co-Cultivation System

**DOI:** 10.3390/mi11030305

**Published:** 2020-03-14

**Authors:** Kae Sato, Momoko Maeda, Eriko Kamata, Sayaka Ishii, Kanako Yanagisawa, Kenji Kitajima, Takahiko Hara

**Affiliations:** 1 Department of Chemical and Biological Sciences, Faculty of Science, Japan Women’s University, Bunkyo, Tokyo 112-8681, Japan; 2 Stem Cell Project, Tokyo Metropolitan Institute of Medical Science, Setagaya, Tokyo 156-8506, Japan; 3 Graduate School of Medical and Dental Sciences, Tokyo Medical and Dental University, Bunkyo, Tokyo 113-8510, Japan; 4 Graduate School of Tokyo Metropolitan University, Hachioji, Tokyo 192-0397, Japan

**Keywords:** embryonic stem cells, hematopoiesis, OP9, microfluidic device

## Abstract

A microfluidic co-culture system, consisting of mouse embryonic stem cells (mESCs)/OP9 cells, was evaluated as a platform for studying hematopoietic differentiation mechanisms in vitro. mESC differentiation into blood cells was achieved in a microchannel that had the minimum size necessary to culture cells. The number of generated blood cells increased or decreased based on the nitric oxide (NO) donor or inhibitor used. Conditioned medium from OP9 cell cultures also promoted an increase in the number of blood cells. The number of generated blood cells under normal medium flow conditions was lower than that observed under the static condition. However, when using a conditioned medium, the number of generated blood cells under flow conditions was the same as that observed under the static condition. We conclude that secreted molecules from OP9 cells have a large influence on the differentiation of mESCs into blood cells. This is the first report of a microfluidic mESC/OP9 co-culture system that can contribute to highly detailed hematopoietic research studies by mimicking the cellular environment.

## 1. Introduction

In mice, hematopoietic stem cells (HSCs) first appear from the hemogenic endothelium, a specialized subset of vascular endothelial cells between E10.5 and E11.5 in the aorta-gonad-mesonephros region of the embryo [1]. Blood flow and shear stress-dependent nitric oxide (NO) play a fundamental role in the emergence and maintenance of HSCs and progenitors [2,3,4,5,6,7]. Treatment with NO synthase (NOS) inhibitors has been reported to reduce intravascular hematopoietic clusters in mouse and zebrafish embryos [2,3]. Mutant mouse and zebrafish embryos lacking heartbeats and, therefore, exhibiting reduced blood flow also displayed reductions in intravascular hematopoietic clusters [2,3].

The role of blood flow in regulating hematopoietic stem cell fate remains poorly characterized, although considerable research efforts have been focused in this area. To elucidate the underlying mechanism, in vitro cell-based experiments that simulate in vivo experimental conditions have been performed [3,5,7]. Currently, there are three standard methods for the induction of hematopoietic differentiation from mouse embryonic stem cells (mESCs) [8], namely, the embryoid body formation method [3], direct differentiation methods, in which a monolayer culture is grown in an extracellular-matrix-coated dish [5], and a co-culture method using mESCs with feeder cells, such as a bone marrow stromal cell line [9,10,11].

The microenvironment surrounding stem cells is important for the differentiation of stem cells into a specific lineage. The OP9 stromal cell line was established from a mouse bone marrow stromal cell with a macrophage colony stimulating factor (M-CSF) gene mutation, resulting in a lack of M-CSF production from the stromal cell. OP9 cells promote hematopoietic differentiation into all types of blood cells, such as erythrocytes, macrophages, granulocytes, megakaryocytes, and B-lineage cells [10,12]. We hypothesized that the integration of the OP9 system into a microbioreactor to mimic the vasculature might produce a viable tool for the study of HSC development.

Three types of bioreactors for hematopoietic cell culture studies have been reported so far: a parallel plate bioreactor system with glass slides [5], a cone-and-plate device with a 95 cm^2^ cell culture dish [3], and a commercially available microchannel (width × depth × length: 17 mm × 0.4 mm × 3.8 mm) provided by ibidi GmbH (Gräfelfing, Germany) [6]. However, the greater dimensions of the reactor channels compared to those of the mouse embryonic aorta require a large number of cells. Furthermore, real-time observation is difficult in the cone-and-plate device. In order to address these issues, we focused on a microfluidic device as a new cell culture tool that can yield complex microscale structures with well-controlled parameters to mimic organ structures and the physical environment in the body [13,14,15,16,17,18,19,20,21,22,23,24]. The advantages of the microfluidic cell culture system for hematopoietic development research include low cell consumption, a small channel size closer to that of the mouse embryonic aorta, continuous perfusion culture, and easy observation.

The aim of the current study is the integration of the OP9 system into a microfluidic device by optimization of microchannel depth, seeding density of OP9 cells and mESCs, and culture days. By using this device, we evaluated the effects of NO treatment, medium flow, and conditioned medium treatment on mESC differentiation to blood cells.

## 2. Materials and Methods 

### 2.1. Device Fabrication

**Figure 1:**
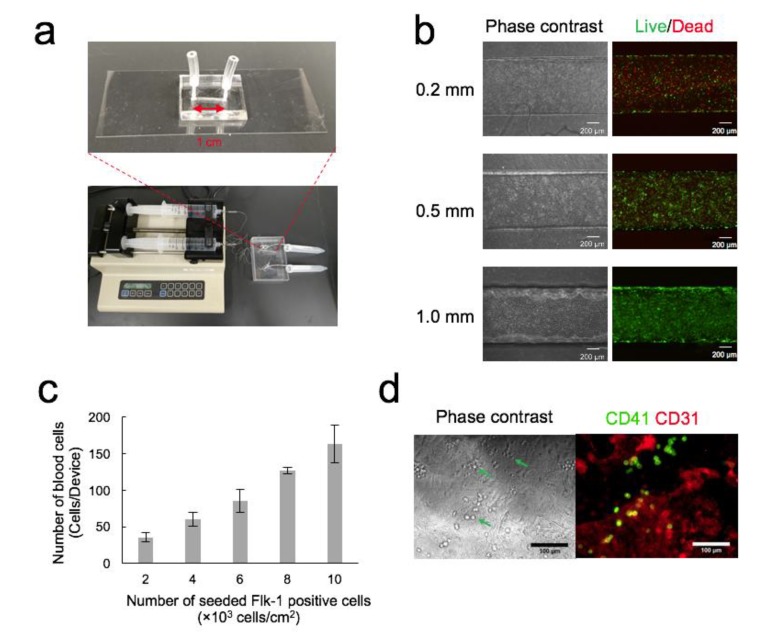
Optimization of cell seeding density. (**a**) Photograph of a microdevice and a syringe pump. (**b**) Live/dead images of OP9 cells after 4-day culture in the microdevice. Channel depth is 0.2, 0.5, or 1.0 mm. (**c**) The number of blood cells produced in the channel. Flk-1^+^ cells were seeded at a density ranging from 2 × 10^3^ cells/cm^2^ to 10 × 10^3^ cells/cm^2^. Mean ± SD, *n* = 3. (**d**) Phase-contrast and immunofluorescence images of the blood and endothelial cells. Arrowheads indicate blood cells. Immunofluorescence staining was performed for the hematopoietic marker CD41, which is expressed on all hematopoietic stem and progenitor cells in the early embryo and the endothelial cell marker CD31.

Polydimethylsiloxane (PDMS) devices (Figure 1a) were fabricated by molding as previously described [25,26,27]. The degassed PDMS prepolymer was poured onto a master with an I-shaped channel structure (depth × width × length, 0.2 mm, 0.5 mm, or 1 mm × 1 mm × 10 mm) to a thickness of 4 mm and baked at 65 °C for 1 h. The PDMS replica was peeled off from the master, mounted on a glass slide (26 mm × 76 mm), and baked again at 100 °C for 1 h. Through-holes were made at either end of the channel with a 1.0 mm biopsy punch. The PDMS replica was bonded to a glass cover slip after plasma treatment (100 W, 35 s) and baked at 100 °C for 30 min. Each hole was connected to a polytetrafluoroethylene (PTFE) tube (0.46 mm i.d. (inside diameter), 0.92 mm o.d. (outside diameter), 10 mm long; Nichias, Tokyo, Japan). The PTFE tube was glued to the hole with the PDMS prepolymer and baked at 100 °C for 1 h.

At one end of the channel, the PTFE tube was connected to a PFA capillary (0.3 mm × 0.5 mm × 800 mm; Iwase, Kanagawa, Japan) via a bubble trap and fabricated as reported previously [28,29]. Briefly, the trap was composed of two TYGON tubes (8 mm length, 0.79 mm i.d., and 2.38 mm o.d.) inserted into either end of a TYGON tube (10 mm length, 2 mm i.d., and 4 mm o.d.). The other end of the PFA capillary was connected to a syringe with a 22G Kel-F (CTFE) hub with the needle removed (KF722, GL Sciences, Tokyo, Japan). At the other end of the channel, the PTFE tube was connected to a TYGON tube (80 mm length, 0.79 mm i.d., and 2.38 mm o.d.). The PDMS devices were packed into heat-sealed paper/plastic pouches and then sterilized by autoclaving and heating.

### 2.2. Preparation of mESCs

mESCs were cultured as previously described [9]. E14tg2a mESCs were cultured in 0.1% gelatin-coated 60 mm dishes for 2 days with a culture medium consisting of KnockOut DMEM (Thermo Fisher Scientific, Waltham, MA, USA) supplemented with 0.1 mM 2-mercaptoethanol (Sigma-Aldrich, St. Louis, MO, USA), 1 mM sodium pyruvate (Thermo Fisher Scientific), 1 × MEM non-essential amino acids (NEAA, Thermo Fisher Scientific), 2 mM L-glutamine (Thermo Fisher Scientific), 1000 unit/mL ESGro (EMD Millipore, Billerica, MA, USA), 1 × penicillin/streptomycin (Thermo Fisher Scientific), and 15% fetal bovine serum (FBS, Thermo Fisher Scientific). Cells were detached by treatment with Accumax (Innovative Cell Technologies, San Diego, CA, USA) on day 2.

To induce differentiation, embryonic stem cells (ESCs; 3 × 10^4^ cells) were plated onto confluent OP9 cells in a 60 mm dish with the OP9 medium α-MEM (Thermo Fisher Scientific) supplemented with 2.2 g/L NaHCO_3_ (FUJIFILM Wako Pure Chemical, Osaka, Japan), 1 × NEAA, 2 mM L-glutamine, 1 × penicillin/streptomycin, and 20% FBS. The medium was replaced on day 3. Six days after seeding, the ESCs were washed twice with phosphate-buffered saline (PBS(−)), collected using Accumax, and frozen in CellBanker (Zenoaq, Fukushima, Japan) at −80 °C. The differentiated and frozen ESCs were thawed and collected by mild pipetting, and then stained with PE anti-mouse CD309 (VEGFR2, Flk-1; BioLegend, San Diego, CA, USA) to be analyzed with a FACSAriaIII cell sorter (BD Biosciences, Franklin Lakes, NJ, USA). The collected Flk-1^+^ cells (including hemogenic endothelial cells) were introduced into a microchannel as described in the following section.

### 2.3. Microfluidic Cell Culture and Differentiation

The microfluidic channel was coated with 0.1% gelatin (FUJIFILM Wako Pure Chemical) at 37 °C for 30 min or 0.1 mg/mL fibronectin (Corning, Corning, NY, USA, or FUJIFILM Wako Pure Chemical) at 4 °C for 16 h. After being washed with a fresh medium, the OP9 cell suspension was introduced into the microfluidic channel (3 × 10^4^ cells/cm^2^). The device was wrapped with a wet lint-free wiper (BEMCOT M-1; Asahi Kasei, Tokyo, Japan) to prevent desiccation, and this was incubated under static conditions in a 5% CO_2_ incubator at 37 °C for 2 days with the OP9 medium. Next, Flk-1^+^ cells isolated by FACS were seeded on OP9 cells in the microfluidic channel (0.2–1.0 × 10^4^ cells/cm^2^) and incubated under static conditions in a 5% CO_2_ incubator at 37 °C in the OP9 medium. After 12 or 24 h, fluid shear stress was applied using a syringe pump (KDS230; KD Scientific, Holliston, MA, USA, or CX07229; Chemyx, Stafford, TX, USA) with a 1 or 5 mL syringe (SS-01T or SS-05SZ, respectively; Terumo, Tokyo, Japan). The flow rates used were 200 µL/h (shear stress, *τ* = 3.3 × 10^−3^ dyn/cm^2^). The syringe pump was programmed to run in a continuous one-directional infusion flow mode or in a bidirectional flow mode with 6 min infusion and 6 min withdraw in turn. The medium in static cultures was changed 24 h after seeding. The generated blood cells were counted manually 2 days after seeding of the Flk-1^+^ cells. Non-adherent round-shaped cells were identified as blood cells.

### 2.4. Cell Viability Assay

The cell viability assay was performed using two fluorescent dyes (LIVE/DEAD Viability/Cytotoxicity assay kit; Thermo Fisher Scientific). Calcein AM (2 μM) and ethidium homodimer (4 μM) in PBS(+) were incubated with the cells in the channel for 30 min at 37 °C under 5% CO_2_ and then rinsed off with PBS(+).

### 2.5. Immunostaining

Cells cultured in the channels were immunostained for CD41 and CD31, which are a transient marker of fetal HSC and an adhesion molecule expressed on endothelial cells, respectively. Briefly, cells were washed with 60 µL PBS(+) three times for 2 min each, fixed with 60 µL 4% paraformaldehyde at 4 °C for 10 min, washed with 60 µL PBS(+) (3 × 2 min washes), and then incubated with 60 µL of 1:250 dilution of Alexa Fluor 488 anti-mouse CD41 antibody (133908, BioLegend) and Alexa Fluor 594 anti-mouse CD31 antibody (102520, BioLegend) in PBS, for 2 h at 4 °C. The cells were then washed with PBS(+) three times for 2 min each. 

### 2.6. Treatment of Cells with Activators and Inhibitors of the NO Pathway

To determine whether activators and inhibitors of the NO pathway could regulate the differentiation of Flk-1^+^ cells into blood cells, Flk-1^+^ cells were incubated with 1, 5, 10, and 20 µM of the NO donor *S*-nitroso-*N*-acetyl-DL-penicillamine (SNAP, FUJIFILM Wako Pure Chemical), 10 µM of the nonspecific NOS inhibitor *N*ω-nitro-L-arginine methyl ester hydrochloride (L-NAME; Dojindo, Kumamoto, Japan), or 10 µM of the NO scavenger 2-(4-Carboxyphenyl)-4,4,5,5-tetramethylimidazoline-1-oxyl-3-oxide (Carboxy-PTIO; Dojindo). In the case of simultaneous addition of SNAP and Carboxy-PTIO, each concentration was 5 µM. The NO donor or inhibitors of the NO pathway were added to the medium 24 h after Flk-1^+^ cell seeding. In the case of the control sample, which received no NO-related reagent, the medium was replaced 24 h after seeding.

### 2.7. Treatment of Cells with Conditioned Medium

The conditioned medium was collected from a dish with OP9 cells after a 48 or 72 h incubation, and a fresh supernatant was used. Flk-1^+^ cells suspended in the conditioned medium (48 h incubation) were seeded on OP9 cells in the channel, followed by incubation under static conditions in a 5% CO_2_ incubator at 37 °C. After 24 h, fluid shear stress was applied using a syringe pump (200 µL/h, 3.3 × 10^−3^ dyn/cm^2^, one-directional infusion flow mode) with the conditioned medium (72 h incubation). In the case of the static culture, the conditioned medium (72 h incubation) was replaced 24 h after seeding.

### 2.8. Microscopy

Fluorescence images were obtained using an IX71 or IX83 Microscope (Olympus, Tokyo, Japan) equipped with a 100 W high-pressure mercury lamp and a cooled CCD camera, ORCA-R2 (Hamamatsu Photonics, Hamamatsu, Japan). A dichroic mirror block (U-MWIG3, excitation 530–550 nm, emission > 575 nm; Olympus) was used for the observation of CD31 antibodies and ethidium homodimers. For the observation of calcein-AM and CD41 antibodies, another dichroic mirror block (U-MNIBA3, excitation 470–495 nm, emission 510–550 nm; Olympus) was used.

### 2.9. Data Analysis

All data were analyzed by averaging the values obtained from three microfluidic devices. Student’s *t*-tests or one-way analysis of variance (ANOVA) and Tukey’s test were performed on the data sets as indicated. 

## 3. Results

### 3.1. Optimization of OP9 Cell Seeding Density in Channel

Figure 1a shows the setup of the microdevice and the syringe pump used in this study. The cell culture surface area in the channel was 10 mm^2^. OP9 cells were used as feeder cells in the channel for the differentiation of Flk-1^+^ cells into blood cells. In this experiment, OP9 cells were grown to confluence in the channel before the seeding of Flk-1^+^ cells. Moreover, OP9 cells were required to be in the culture without medium change for 2 days until Flk-1^+^ cells were differentiated into blood cells. To optimize the seeding and culture conditions, OP9 cells were seeded in the channel (0.2, 0.5, and 1.0 mm deep) with varying initial cell seeding densities followed by staining with live/dead assay reagents every 24 h.

The initial cell density at 30 × 10^4^ cells/cm^2^ was not suitable for all channels. Although the cells remained attached to the surface of the channel 3 h after seeding, a sizeable fraction of the cell population gradually detached to form aggregates. When the initial cell density was 15 × 10^4^ cells/cm^2^, the cells grew to confluence and were viable 24 h after seeding, while the proportion of dead cells increased after 72 h.

The cells that seeded at the initial cell density of 3 × 10^4^ cells/cm^2^ grew rapidly to confluence and were viable in all channels 48 h after seeding. Dead cells were observed 96 h after seeding in 0.2 mm and 0.5 mm deep channels, while the cell layer in the 1.0 mm deep channel was confluent and healthy (Figure 1b). Thus, we decided to use a channel with 1.0 mm depth, 3 × 10^4^ cells/cm^2^ initial seeding density, and a two-day culture prior to the seeding of Flk-1^+^ cells for subsequent experiments.

### 3.2. Optimization of Seeding Density of Flk-1^+^ Cells

The number of blood cells increased with the seeding density of Flk-1^+^ cells (Figure 1c). When the seeding density was 10 × 10^3^ Flk-1^+^ cells/cm^2^, the number of blood cells was 163.5 ± 25.5 cells/channel (*n* = 3). We set the seeding density at 10 × 10^3^ cells/cm^2^ for subsequent experiments. The blood and endothelial cells stained with CD41 and CD31 antibodies are shown in Figure 1d. All cells were alive (Figure S1).

### 3.3. Effects of NO Donor and NO Pathway Inhibitors

NO signaling contributes to the induction of HSC formation [2,3]. Inhibition of NO production with a NOS inhibitor has been shown to cause a marked decrease in HSCs and hematopoietic progenitors both in vivo and in vitro, while a NO donor led to a dose-dependent increase [2]. To determine the role of NO in the OP9 system, Flk-1^+^ cells were treated with multiple concentrations of NO donor or inhibitor.

**Figure 2:**
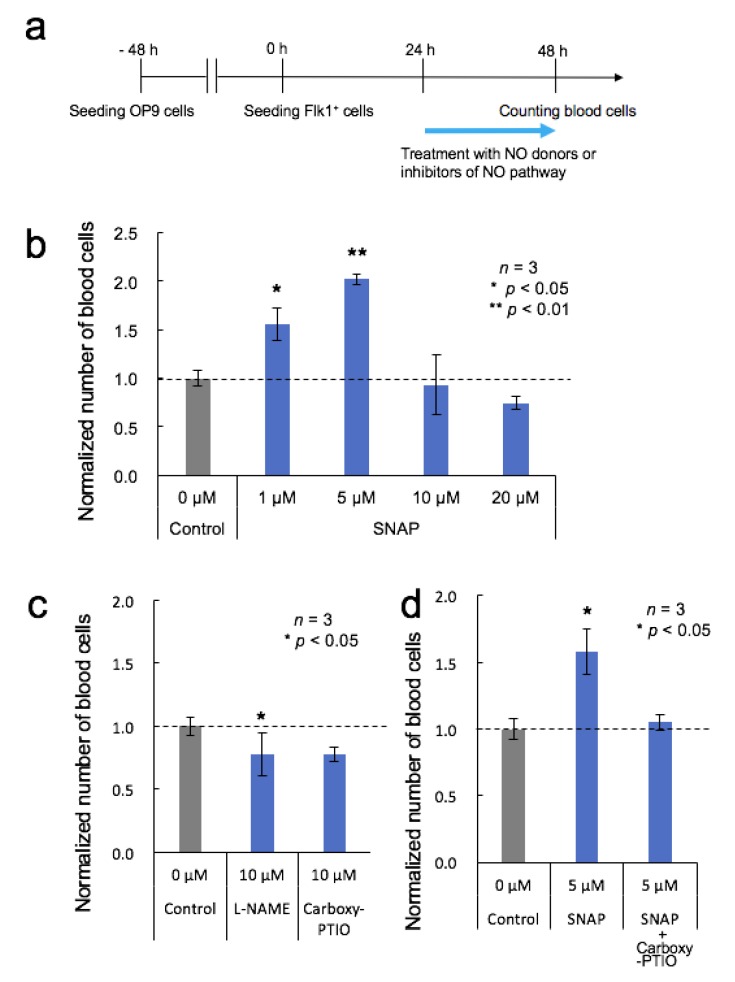
Effects of the nitric oxide (NO) donor and NO pathway inhibitors on blood cell generation. (**a**) Timeline of the differentiation procedure and NO stimulation. (**b**) The number of blood cells arising from Flk-1^+^ cells untreated or treated with SNAP. Mean ± SD, one-way ANOVA, *: *p* < 0.05, **: *p* < 0.01, *n* = 3. (**c**) The number of blood cells arising from Flk-1^+^ cells untreated or treated with L-NAME or 2-(4-Carboxyphenyl)-4,4,5,5-tetramethylimidazoline-1-oxyl-3-oxide (Carboxy-PTIO). Mean ± SD, significant versus control, *t*-test, *: *p* < 0.05, *n* = 3. (**d**) The number of blood cells arising from Flk-1^+^ cells untreated or treated with SNAP or the combination of SNAP and Carboxy-PTIO. Mean ± SD, significant versus control, *t*-test, *: *p* < 0.05, *n* = 3.

Figure 2a shows the timeline of the NO-related experiments. The NO donor or the inhibitor of the NO pathway was added to the medium 24 h after Flk-1^+^ cell seeding. Figure 2b shows the number of blood cells normalized to the number of untreated control cells 48 h after Flk-1^+^ cell seeding. Although NO production by 1 µM and 5 µM SNAP stimulation led to an increase in the number of blood cells, as expected, 10 µM and 20 µM SNAP did not lead to the increase, suggesting that these higher concentrations were beyond the appropriate range for blood cell generation. 

Similarly, inhibition of NO signaling with L-NAME or Carboxy-PTIO resulted in a reduction of the number of generated blood cells compared to that in control cells (Figure 2c). In addition, inhibition with Carboxy-PTIO resulted in a significant reduction of the SNAP-induced increase in blood cell generation to levels with almost the same number as those in control cells (Figure 2d). Taken together, our results agree with previous reports [2,3], and we concluded that NO production might support blood cell generation in the OP9 system.

### 3.4. Effects of Medium Flow

**Figure 3:**
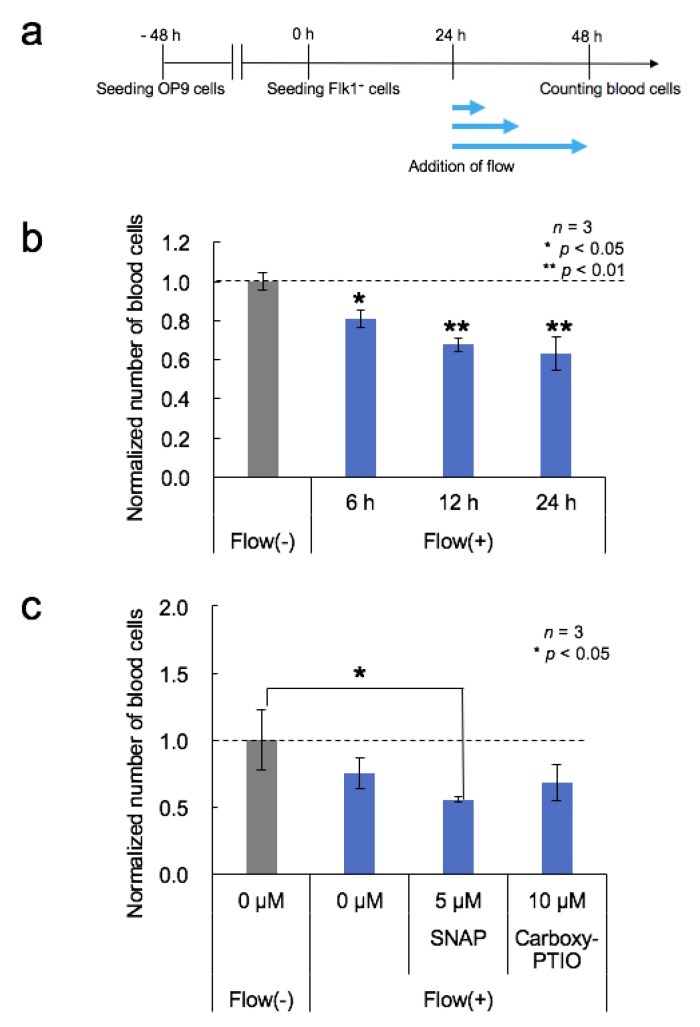
Effects of medium flow. (**a**) Timeline of the differentiation procedure and flow stimulation. (**b**) The number of blood cells arising from Flk-1^+^ cells under flow conditions with different durations. Mean ± SD, one-way ANOVA, *: *p* < 0.05, **: *p* < 0.01, *n* = 3. (**c**) Effects of fluid flow stress, NO donor, and NO pathway inhibitors on blood cell generation. The number of blood cells arising from Flk-1^+^ cells exposed to fluid shear stress of 3.3 × 10^−3^ dyn/cm^2^ and SNAP or Carboxy-PTIO stimulation. Mean ± SD, one-way ANOVA, *: *p* < 0.05, *n* = 3.

Within the embryo at E10.5, vascular shear stress levels have been estimated to be approximately 5 dyn/cm^2^ [3,30]. Based on these reports, OP9 cells were cultured in the channel under static conditions for three days and then exposed to fluid shear stress in a step-wise fashion every 0.5 h for 5 h from 0 to 5 dyn/cm^2^ (10 steps, 0.5 dyn/cm^2^ each). As a result, OP9 cells could not be alive under vascular shear stress levels. OP9 cells were gradually detached at 1 dyn/cm^2^ and most cells in the channel were dead at 5 dyn/cm^2^. According to these results, shear stress conditions below 1 dyn/cm^2^ were examined. Cells exposed to shear stress at 8.3 × 10^−5^ or 3.3 × 10^−3^ dyn/cm^2^ grew well, whereas those exposed to shear stress at 2.1 × 10^−2^ dyn/cm^2^ were detached. Therefore, we employed low shear stress in the following experiments. Figure 3a shows the timeline of flow culture-related experiments. Flk-1^+^ cells were seeded on OP9 cells in the channel, cultured under static conditions for 24 h to allow cell attachment, and then exposed to shear stress at 3.3 × 10^−3^ dyn/cm^2^.

The effect of increased duration of fluid flow was evaluated. Shear stress was applied at 3.3 × 10^−3^ dyn/cm^2^, and the number of generated blood cells decreased with increasing flow duration (Figure 3b). To eliminate the possibility that Flk-1^+^ cells were washed away by the flowing medium, we examined whether the cells remained in the channel during flow culture. Flk-1^+^ cells, expressing mCherry, were used instead of normal ESCs, and the densities of mCherry-expressing cells were comparable at 3 h (before flow culture) and at 48 h (after 24 h of flow culture) after seeding of the cells (Figure S2). In addition, 48 h after the cells were seeded, the densities of mCherry-expressing cells under all flow conditions were similar to those under static conditions. Based on these observations, we concluded that Flk-1^+^ cells were not washed away by the flowing medium, and believe the reason behind the reduction in the number of blood cells with flow duration was that soluble signaling molecules secreted by the cells were washed away by the medium flow, and the differentiation into blood cells was reduced by the insufficient signaling molecules. 

Next, the effects of medium flow on the NO donor/inhibitor-treated cells were examined (Figure 3c). Five micromolar SNAP stimulations led to an increase in the number of blood cells under static conditions (Figure 2b), whereas both SNAP stimulation and shear stress at 3.3 × 10^−3^ dyn/cm^2^ (200 µL/h) led to a decrease in the number of blood cells (Figure 3c). Since shear stress activates NO production, this result suggests that the experimental conditions might have been beyond the appropriate range of NO concentration for blood cell generation. Additionally, the number of blood cells generated with the NO signaling inhibitor, 10 µM Carboxy-PTIO, and shear stress at 3.3 × 10^−3^ dyn/cm^2^ was almost the same as that in control cells. Therefore, the number of blood cells generated with 10 µM Carboxy-PTIO and shear stress was higher than that with 10 µM Carboxy-PTIO alone (Figure 2c). This result suggests that shear stress-induced NO production might counteract the effects of the NO pathway inhibition.

### 3.5. Effects of Fluid Flow Stress and Conditioned Medium

**Figure 4:**
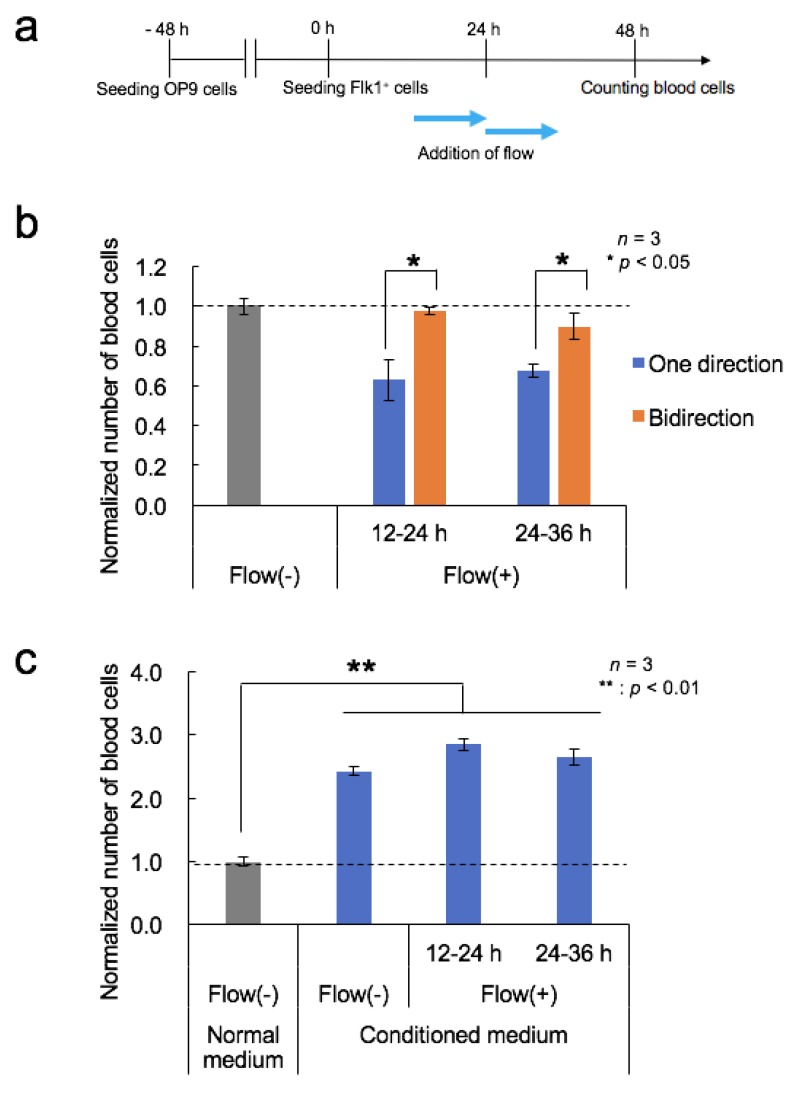
Effects of fluid flow stress and conditioned medium. (**a**) Timeline of the differentiation procedure and flow stimulation. (**b**) The number of blood cells generated from Flk-1^+^ cells cultured under unidirectional or bidirectional flow from 12 to 24 h or 24 to 36 h after seeding of cells. Mean ± SD, *t*-test, bidirectional versus one direction, *: *p* < 0.05, *n* = 3. (**c**) The number of blood cells arising from Flk-1^+^ cells in the conditioned medium. Mean ± SD, one-way ANOVA, **: *p* < 0.01, *n* = 3.

We hypothesized two possibilities for the reduced differentiation efficiency in Figure 3b: (1) fluid flow stress itself reduced the differentiation, or (2) the secreted soluble signaling molecules were washed away. To identify the key reason, fluid flow stress was applied with a medium containing enough soluble signaling molecules to differentiate the cells into blood cells. The syringe pump was programmed to run in bidirectional flow mode with a 6-min infusion and 6-min withdrawal in order to prevent washing away of the soluble signaling molecules. The effects of medium flow timing were also examined (Figure 4a). As a result, the number of blood cells generated under the bidirectional flow conditions was larger than that under unidirectional flow conditions, both from 12 to 24 h and from 24 to 36 h and was similar to the number generated under static conditions (Figure 4b). Thus, we concluded that fluid flow stress itself did not reduce the differentiation, and an insufficient supply of soluble signaling molecules secreted by the cells affected blood cell generation.

Next, the effects of the conditioned medium from the OP9 culture were examined. The number of generated blood cells increased when using the conditioned medium in either static- or flow-conditioned cultures (Figure 4c). The number of blood cells cultured under static conditions with the conditioned medium was two times larger than that with the normal medium (*p* = 6.8 × 10^−4^). The number of blood cells obtained from the flow experiments was similar to that in the static with the conditioned medium. 

To investigate the effects of the conditioned medium in more detail, we filtered the medium to remove high molecular weight molecules (10 kDa cutoff). The filtered conditioned medium reduced the number of generated blood cells compared to that generated in the unfiltered conditioned medium (Figure S3). These results suggest that the effective soluble factor(s) are larger than 10 kDa in size.

## 4. Conclusions

In summary, a microfluidic co-culture system consisting of mESCs on OP9 cells was evaluated as a viable platform for studying hematopoietic differentiation mechanisms. Seeding densities of OP9 and Flk-1^+^ cells from mESCs were adequately optimized. mESC differentiation into blood cells was achieved in a microchannel that had the minimum size necessary to culture the cells and closely mimicked the size of the mouse embryonic aorta [31]. Since the microchannel has a mimicked size of the mouse embryonic aorta, it might have a cellular biochemical microenvironment that is similar to the mouse embryo compared to a conventional cell culture dish. The number of the generated blood cells was increased by NO-dependent stimulation. These results agree with those of previous studies [2,3]. OP9 cells could not be alive under vascular shear stress levels. Therefore, we could only examine the influence of low shear stress on the differentiation of Flk-1^+^ cells into blood cells. Fluid flow stress did not promote differentiation in this study. On the other hand, new knowledge that the conditioned medium from OP9 cell cultures promoted an increase in the number of blood cells was gained. From the above results, we concluded that secreted molecules from OP9 cells had a large influence on the differentiation of Flk-1^+^ cells into blood cells. The microfluidic mouse embryonic stem cell/OP9 co-cultivation system realized low cell consumption, a small channel size closer to that of the mouse embryonic aorta, a continuous perfusion culture, and an easy observation. This system will be helpful for hematopoietic development research.

## Supplementary Materials

The following are available online at https://www.mdpi.com/2072-666X/11/3/305/s1, Figure S1: Phase contrast and live/dead images of the blood cells and OP9 cells, Figure S2: Observation of mCherry ESCs before and after flow culture, Figure S3: Effects of filtered conditioned medium.
