# Supplementary material for: Nitric Oxide and a Conditioned Medium Affect the Hematopoietic Development in a Microfluidic Mouse Embryonic Stem Cell/OP9 Co-Cultivation System
Source: Micromachines (Basel). 2020 Mar 14;11(3):305. doi: 10.3390/mi11030305 (PMC7143789; doi:10.3390/mi11030305)
Supplement: Supplementary file 1 [file micromachines-11-00305-s001.pdf]

micromachines

Supplementary Materials:

Nitric Oxide and a Conditioned Medium Affect the Hematopoietic Development in a Microfluidic Mouse Embryonic Stem Cell/OP9 Co-Cultivation System

Kae Sato, Momoko Maeda, Eriko Kamata, Sayaka Ishii, Kanako Yanagisawa, Kenji Kitajima and Takahiko Hara

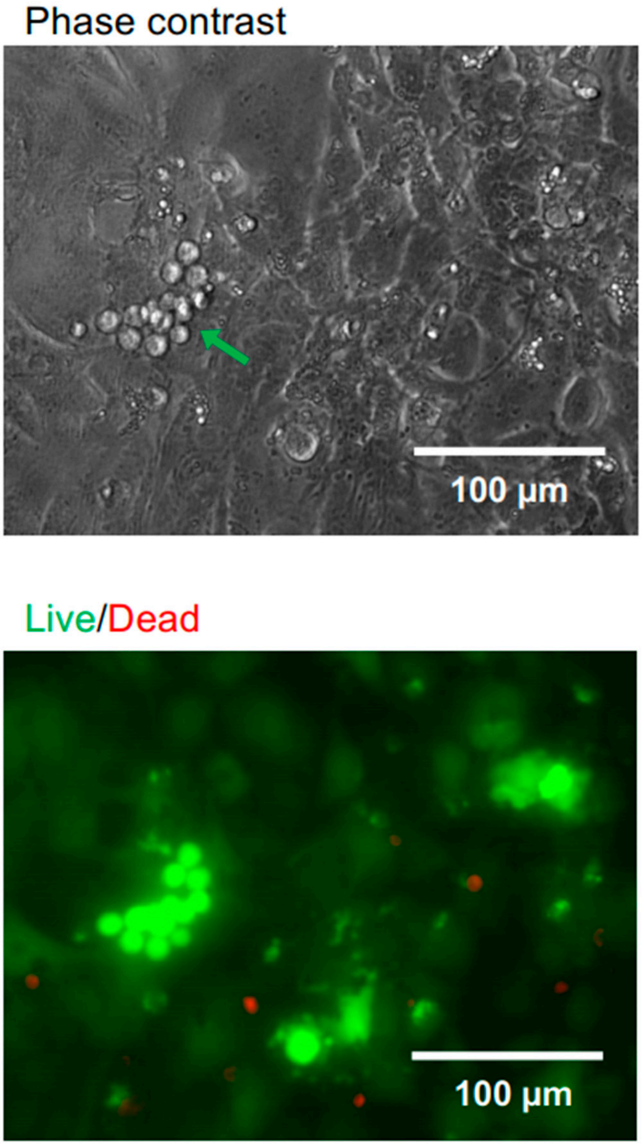

Figure S1. Phase contrast and live/dead images of the blood cells and OP9 cells. Arrowheads indicate blood cells. Live/dead assay staining indicated that most of cells within the microfluidic channel were alive.

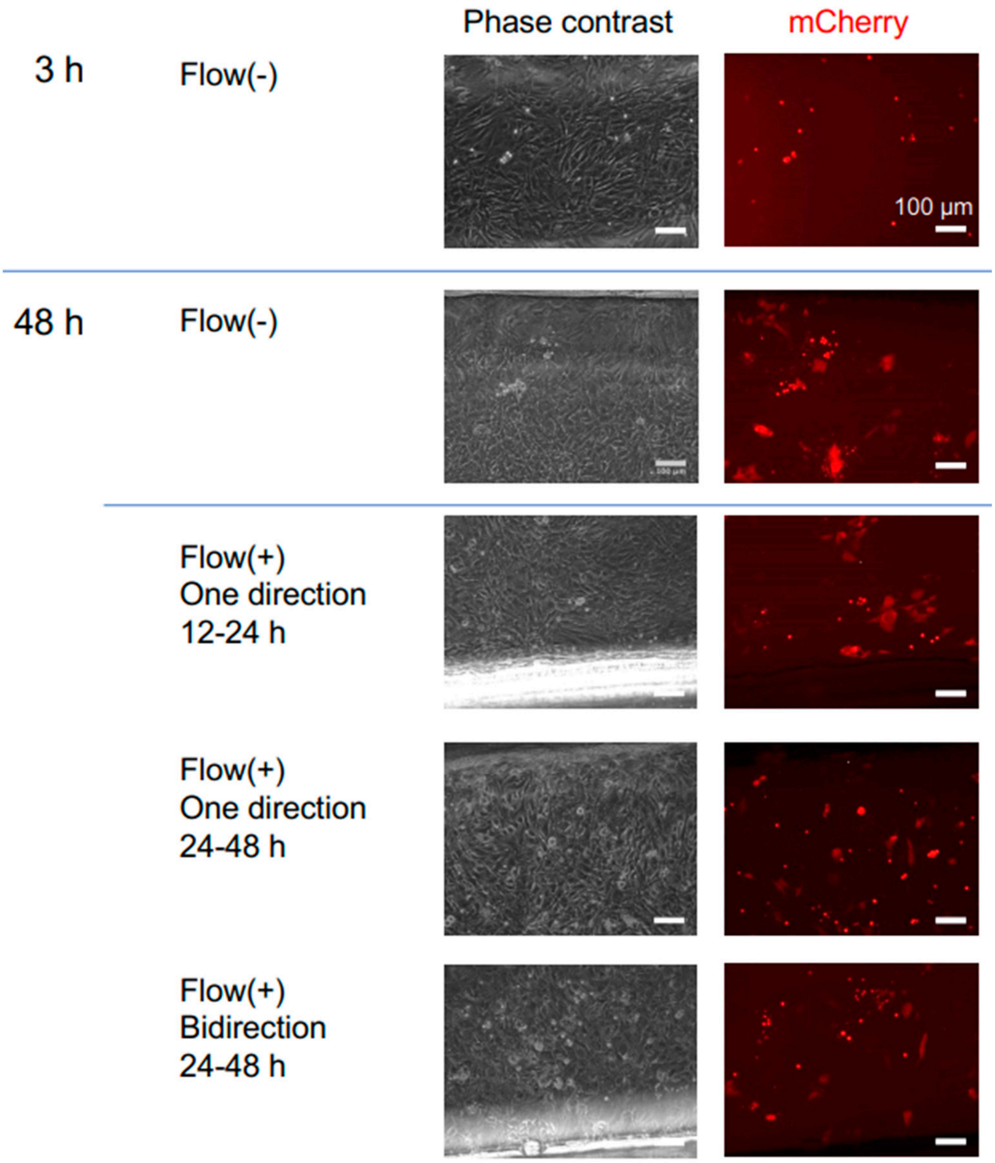

Figure S2. Observation of mCherry embryonic stem cells (ESCs) before and after flow culture. To visualize Flk-1+ cells, dox-inducible mCherry ESCs (A2lox.cre ES) were used instead of E14tg2aESCs. Cell culture protocol was the same as the standard protocol except for addition of doxycycline. Flk-1+ cells from dox-inducible mCherry ESCs were seeded on OP9 cells in the channel. After incubation, the cells were cultured under fluidic or static conditions. Cells were cultured under 4 conditions: static condition, unidirectional flow conditions from 12–24 h, unidirectional flow conditions from 24–48 h, and bidirectional flow conditions from 24–48 h. The status of the cells was observed with a microscope 3 and 48 h after seeding.

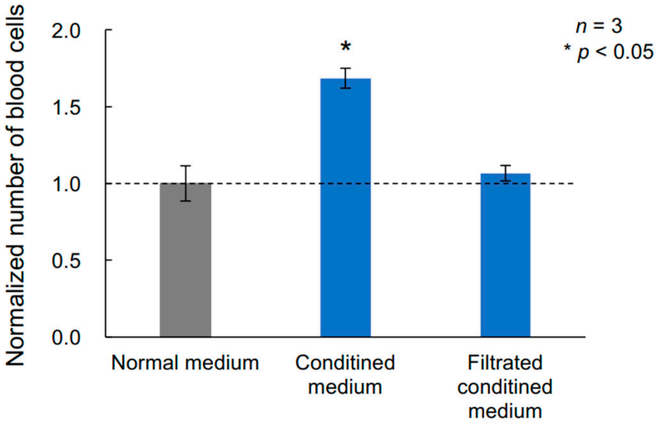

Figure S3. Effects of filtered conditioned medium. The number of blood cells generated in normal, conditioned, and filtered conditioned medium are shown. Filtered conditioned medium was prepared by filtering conditioned medium through a disposable centrifugal ultrafiltration device (molecular weight cutoff 10 kDa; Amicon Ultra-0.5 mL Centrifugal Filters Ultracel-10K; EMD Millipore, Billerica, MA, USA). The filtered conditioned medium was supplemented with 20% FBS and used as Flk-1+ cell culture medium. The medium was replaced 5 and 22 h after being seeded with Flk-1+ cells.

© 2020 by the authors. Submitted for possible open access publication under the terms and conditions of the Creative Commons Attribution (CC BY) license (http://creativecommons.org/licenses/by/4.0/).
